# Indoleamine 2,3-Dioxygenase Is Involved in Interferon Gamma’s Anti-BKPyV Activity in Renal Cells

**DOI:** 10.3390/v12080865

**Published:** 2020-08-07

**Authors:** Tony Fiore, Elodie Martin, Véronique Descamps, Etienne Brochot, Virginie Morel, Lynda Handala, Fatima Dakroub, Sandrine Castelain, Gilles Duverlie, François Helle, Catherine François

**Affiliations:** 1 UR4294, Infectious Agents, Resistance and Chemotherapy, University Health Research Center, University of Picardie Jules Verne, F-80054 Amiens, France; toni.fiore37@gmail.com (T.F.); e.martin2711@gmail.com (E.M.); descamps.veronique@chu-amiens.fr (V.D.); brochot.etienne@chu-amiens.fr (E.B.); morel.virginie@chu-amiens.fr (V.M.); l.handala@gmail.com (L.H.); fatimadakroub20@gmail.com (F.D.); castelain.sandrine@chu-amiens.fr (S.C.); gilles.duverlie@u-picardie.fr (G.D.); francois.helle@u-picardie.fr (F.H.); 2 Department of Virology, Amiens University Hospital, F-80000 Amiens, France

**Keywords:** BK virus, interferon, indoleamine-2,3-dioxygenase, immunity

## Abstract

Reactivation of BK polyomavirus (BKPyV) infection is frequently increasing in transplant recipients treated with potent immunosuppressants and highlights the importance of immune system components in controlling viral reactivation. However, the immune response to BKPyV in general and the role of antiviral cytokines in infection control in particular are poorly understood. Here, we investigated the efficacy of interferons (IFN) alpha, lambda and gamma with regard to the BKPyV multiplication in Vero cells. Treatment with IFN-gamma inhibited the expression of the viral protein VP1 in a dose-dependent manner and decreased the expression of early and late viral transcripts. Viral inhibition by IFN-gamma was confirmed in human cells (Caki-1 cells and renal proximal tubular epithelial cells). One of the IFN-stimulated genes most strongly induced by IFN-gamma was the coding for the enzyme indoleamine 2,3 dioxygenase (IDO), which is known to limit viral replication and regulates the host immune system. The antiviral activity induced by IFN-gamma could be reversed by the addition of an IDO inhibitor, indicating that IDO has a specific role in anti-BKPyV activity. A better understanding of the action mechanism of these IFN-gamma-induced antiviral proteins might facilitate the development of novel therapeutic strategies.

## 1. Introduction

The ubiquitous BK polyomavirus (BKPyV) is involved in the development of nephropathy after kidney transplantation. The emergence of this BKPyV-associated nephropathy (BKVAN) [1] appears to be correlated with the clinical introduction of more powerful immunosuppressants [2]. In view of the growing number of immunocompromised or immunosuppressed patients, BKPyV is an increasingly important public health issue. Following an asymptomatic primary infection, the virus settles in the urinary tract and establishes a persistent, subclinical infection there. BKPyV-associated nephropathy is characterized by high-level BKPyV replication in the transplanted kidney’s proximal tubular epithelial cells, which results in cell lysis and denudation of the tubular monolayer. The influx of inflammatory cells into the interstitium leads to tubular atrophy, interstitial fibrosis [3], and thus impaired renal function. BKVAN affects 1% to 10% of kidney transplant patients in the first two years after transplantation [2,4]. Since an effective antiviral drug is not available, the most common treatment approach involves decreasing immunosuppressive therapy to reduce the risk of BKVAN.

Interferons (IFNs) are key antiviral cytokines that act in the body’s first-line defense against infection [5]. The pro-inflammatory cytokine IFN-gamma is the only member of the type II interferon family. It is produced by innate immune cells (natural killer (NK) and antigen presenting cells) and T-cells during the adaptive immune response. Interferon-gamma activates the Janus kinase/signal transducer and transcription activator (JAK-STAT) pathway and thus allows the transcription of IFN-stimulated genes (ISGs)—many of which are involved in the fight against viral infections. Moreover, this antiviral cytokine stimulates the maturation of T and B lymphocytes and increases antibody production. It also enhances the expression of human leukocyte antigen class I and II molecules by macrophages and activates neutrophils and NK cells. However, IFN-gamma’s role in controlling BKPyV infection is poorly documented. Only one study has demonstrated the effect of IFN-gamma on BKPyV [6]. Various IFN-gamma-regulated antiviral mechanisms can be induced in target cells. Interferon-gamma is a potent activator of indoleamine 2,3-dioxygenase (IDO, an enzyme involved in tryptophan catabolism). It is thought that IDO contributes to the host’s innate defenses and regulates the immune response. Since many microbial organisms depend on tryptophan, it has been suggested that the degradation of this amino acid by IDO-expressing innate immune cells is the prime IDO-mediated mechanism of defense against infections [7]. The enzyme’s possible involvement in the control of viral infections has been evidenced by in vitro experiments in which cytomegalovirus (CMV) replication was inhibited by treatment with IFN-gamma [8]. Subsequently, viruses such as herpes simplex virus type 2 [9], measles virus [10] and vaccinia virus [11] have been found to be susceptible to the depletion tryptophan by IDO.

Here, we analyzed the possible role of IDO secreted by IFN-gamma-stimulated cells. Our results suggest that IDO has an antiviral activity on BKPyV in vitro in Caki-1 and RPTE cells.

## 2. Materials and Methods

### 2.1. Cell Cultures

The monkey-derived Vero cell line and the human Caki-1 cell line were obtained from the ATCC (ATCC-CCL-81 and ATCC-HTB-46, respectively) and cultured in Dulbecco’s modified Eagle’s medium (Invitrogen) supplemented with 10% fetal bovine serum. Human renal proximal tubular epithelial cells immortalized with pLXSN-hTERT retroviral transfection (RPTE/TERT1 cells) were obtained from Evercyte and maintained in renal epithelial cell basal medium (REGM CC-3191, Lonza) supplemented with human epidermal growth factor, 0.5% fetal bovine serum, hydrocortisone, epinephrine, insulin, triiodothyronine, transferrin and GA-1000 (REGM SingleQuots^TM^ Supplement Pack, CC-4127, Lonza). All cells were cultured in a humidified environment with 5% CO_2_ at 37 °C.

### 2.2. Antibodies and Reagents

The rabbit recombinant monoclonal anti-IDO antibody (ab211017) was purchased from Abcam. The monoclonal antibody (mAb) for the detection of STAT-1 expression was purchased from Santa Cruz (C-136) and the mAb for the detection of phospho-STAT1 (Tyr701) expression was obtained from Cell Signaling (D4A7). The 3B2 monoclonal anti-BKPyV VP1 antibody, the rabbit anti-mouse IgG (whole molecule) peroxidase-labelled antibody and the mouse anti-rabbit IgG (whole molecule) peroxidase-labelled antibody were obtained from Calbiochem. Alexa 260 Fluor Plus 488-conjugated goat anti-Mouse IgG (H + L) was purchased from Thermo Fisher. Recombinant human IFN-gamma was purchased from Roche (100,000 IU; Switzerland) and recombinant human IFN-alpha and IFN-lambda 1 were obtained from PBL Assay Science. Pyridone-6 and the pharmacological IDO inhibitor IDO5L were obtained from Calbiochem and Fisher Scientific, respectively.

### 2.3. BKPyV Production 

The BKV-pUC19 plasmid (kindly provided by W.J. Atwood, Brown University, USA) was used to produce the BKPyV. The plasmid was obtained from pBKv (34-2) (Dunlop strain, genotype I), as described previously [12], and digested with BamHI (New England Biolabs; 2 U for every 1 µg of DNA) for 4 h at 37 °C to separate the BKPyV genome from the backbone. The DNA was then incubated at 65 °C (to inactivate BamHI) and was transfected into Vero cells using lipofectamine (Invitrogen), according to the manufacturer’s instructions. Cells were cultured until a cytopathic effect was observed (after 4 weeks, typically). Next, the BKPyV was amplified by successive infections of naive cells at a multiplicity of infection (MOI) of one every four days. Extracellular and intracellular viral particles were harvested, extracted with chloroform [13] and filtered at 0.45 µm. The titers of viral stocks were determined by the 50% tissue culture infective dose immunofluorescence method (see below).

### 2.4. Cell Viability Assay

Vero cells were treated with different IFN concentrations in the presence of BKPyV for 72 h. The percentage of viable cells was measured using the CellTiter-glo^®^ luminescent/cell viability assay (Promega, Madison, WI, USA), as described previously [14].

### 2.5. Immunofluorescence Staining

Cells were washed with PBS, fixed with paraformaldehyde (3.7% in PBS) and permeabilized with Triton-X100 (0.5% in cytoskeleton buffer: 10 mM PIPES, 300 mM sucrose, 100 mM NaCl, 3 mM MgCl2, 1 mM EGTA). Infected cells were detected by immunofluorescence staining of VP1. Nuclei were stained with 4′,6-diamidino-2-phenylindole (DAPI). Immunostained cells were observed with a Zeiss Axio Vert.A1 microscope equipped with Colibri 7 LED 298 illumination (Zeiss, Oberkochen, Germany). Fluorescent signals were acquired with an Axiocam 305 color camera (Zeiss). The percentage of infected cells was automatically determined using the QuantIF macro in ImageJ (version 1.52e and Java version 8; freely available online at MDPI.com) [15].

### 2.6. Western-Blot

Total cell proteins were harvested 3 days after infection using radioimmunoprecipitation assay buffer (20 mM HEPES KOH pH 7.4; 150 mM NaCl; 5 mM EDTA; 0.1% SDS; 1% DOC and 1% Triton X-100). Proteins were separated by SDS-PAGE and transferred to nitrocellulose membranes (Bio-Rad, Hercules, CA, USA). Proteins of interest were identified using Immobilon Western Chemiluminescent HRP Substrate (Millipore, Burlington, MA, USA), as recommended by the manufacturer. The primary antibodies were diluted in PBS containing 0.1% Tween, 5% bovine serum albumin and 0.05% azide, and the secondary antibodies were diluted in PBS containing 0.1% Tween and 5% dried milk.

### 2.7. Real-Time PCR

Total cellular RNA was extracted using a RNeasy Mini kit and the “Animal tissues and cells” protocol from Qiagen, according to the manufacturer’s instructions. Total RNA was reverse transcribed using a High Capacity cDNA Reverse Transcription Kit from Life Technologies. The RNA was amplified with TaqMan Universal Master Mix on an ABI 79000HT Sequence Detection System (Life Technologies, Carlsbad, CA, USA) using primers and probe sets for VP1, *IDO1* and the housekeeping gene *GAPDH*. Inhibition of BKPyV was normalized against the results for *GAPDH* and expressed according to the comparative Ct method as the fold induction [16].

### 2.8. Kynurenine Assay

One hundred and fifty microliters of culture supernatant were transferred to a 96-well U-bottom plate. Ten microliters of 30% trichloroacetic acid (Sigma-Aldrich, St-Louis, MO, USA) were added to each well and the plates were incubated for 30 min at 50 °C. The samples were then centrifuged at 2000× *g* for 10 min at room temperature. Next, 100 μL of supernatant were harvested and mixed with 100 μL of freshly prepared Ehrlich’s reagent (2% 4-dimethylaminobenzaldehyde in glacial acetic acid; Fisher Scientific, Hampton, NH, USA). Absorbance at 492 nm was measured using a microplate reader (Tecan, Mannedorf, Switzerland).

### 2.9. Statistical Analysis

Data were expressed as the mean ± standard deviation (SD). Conditions were compared in a two-way analysis of variance with Bonferroni’s correction different. The threshold for statistical significance was set to *p* < 0.05.

## 3. Results

### 3.1. Interferon-Gamma Inhibits BKPyV Multiplication more Potently than IFN-Alpha and IFN-Lambda 1

**Figure 1:**
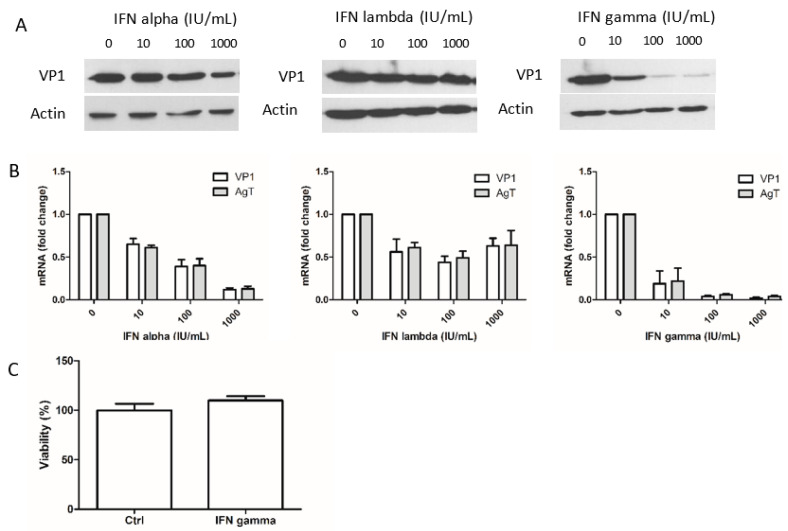
Effects of the three types of interferons (IFN) on BKPyV multiplication. (**A**) Vero cells were pre-incubated for 6 h with different concentrations of IFN-alpha, lambda or gamma and then inoculated for 3 h with BKPyV (multiplicity of infection, MOI = 0.5). Total cellular proteins were extracted 72 h post-infection. The IFN was present throughout the experiment. VP1 and actin proteins were detected using Western blotting with specific antibodies. (**B**) Levels of LTag and VP1 transcripts were determined by qPCR. The results are the mean of three independent experiments performed in duplicate. (**C**) The absence of cytotoxicity in the presence of IFN-gamma was confirmed by the results of a cell viability assay.

To determine the antiviral efficacy of the various types of IFN (IFN-alpha, IFN-lambda 1 or IFN-gamma), we measured the expression of the BKPyV structural protein VP1 in the presence of increasing concentrations of each IFN. To allow the induction of ISGs, Vero cells were incubated with IFN for 6 h before infection. As shown in Figure 1A, VP1 expression was only slightly inhibited by 100 and 1000 IU/mL of IFN-alpha but was strongly inhibited by 10 IU/mL of IFN-gamma. The expression of VP1 was unaffected by IFN-lambda. We also investigated viral RNA transcription by measuring the levels of mRNA for the early large T antigen (LTag) and the late structural protein VP1 three days post-infection. As shown in Figure 1B, early and late viral mRNA were inhibited in much the same manner as VP1 protein expression. The effect of IFN-gamma was not associated with changes in cell viability (Figure 1C). Interferon-lambda had a weak inhibitory effect on viral mRNA on Vero cells. In conclusion, IFN-gamma was the most potent antiviral IFN with regard to the inhibition of BKPyV multiplication.

### 3.2. The Jak-Stat Pathway Is Involved in the Antiviral Effect of IFNs on BKPyV Infection

**Figure 2:**
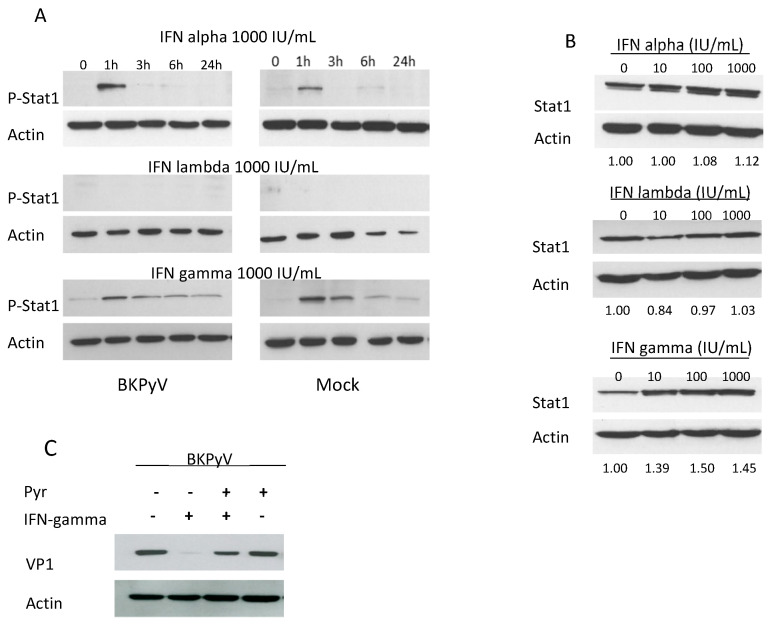
The Janus kinase/signal transducers and activators of transcription (JAK-STAT) pathway is involved in IFN’s effect on BKPyV infection. (**A**) Vero cells were inoculated for 3 h with BKPyV at a MOI of 0.5. Twenty-four hours post-infection, the cells were incubated with 1000 IU/mL of IFN-alpha, -lambda or -gamma. Total cellular proteins were harvested 0, 1, 3, 6 and 24 h after IFN treatment. Phospho-Stat1 (Y 701) and actin proteins were detected by Western blotting. (**B**) Vero cells were pre-incubated for 6 h with different IFNs concentrations and then inoculated for 3 h with BKPyV at a MOI of 0.5. Total cellular proteins were harvested 72 h after infection. The IFN was present throughout the experiment. STAT1 and actin proteins were detected by Western blotting. (**C**) Vero cells were incubated 4 h with 10 IU/mL IFN-gamma in the presence or absence of 10 μM pyridone-6 and then infected for 3 h with BKPyV at a MOI of 0.5. The IFN and pyridone-6 were present throughout the experiment. Three days after infection, total cellular proteins were extracted and VP1 and actin were detected by Western blotting.

The three types of IFN have a common signaling pathway. The Janus kinase/signal transducers and activators of transcription (JAK-STAT) pathway mainly comprises STAT1 and STAT2 heterodimers for IFN-alpha and lambda, and mainly STAT1 homodimers for IFN-gamma. As the three types of IFN had different effects on BKPyV infection, we investigated the JAK-STAT pathway’s role in this context. First, we measured STAT1’s phosphorylation status in Vero cells treated with 1000 IU/mL of the various types of IFN. As shown in Figure 2A, we detected brief STAT1 phosphorylation with IFN-alpha, prolonged phosphorylation with IFN-gamma and no phosphorylation with IFN-lambda. Under these conditions, the presence of BKPyV did not modify STAT1 phosphorylation. We also investigated the expression of STAT1 protein (encoded by an ISG). As shown in Figure 2B, the expression of STAT1 in Vero cells was weakly induced by IFN-alpha, strongly induced with 10 IU/mL of IFN-gamma and not induced by IFN-lambda. This confirmed that ISG induction was correlated to both Stat1 phosphorylation and IFN’s antiviral effects. The JAK-STAT pathway’s involvement was also confirmed by the fact that the JAK inhibitor pyridone-6 restored BKPyV’s infection of Vero cells in the presence of IFN-gamma (Figure 2C). We conclude that IFN-gamma has a potent antiviral effect on BKPyV infection and is associated with a particular STAT1 phosphorylation profile.

To find out which proteins might be involved in IFN-gamma’s antiviral effect on BKPyV, we analyzed the Vero cells’ transcriptomic study with a low-density array. Nine of the 64 genes studied had an induction profile that resembled that of the antiviral effect: moderate induction with IFN-alpha, poor induction with IFN-lambda and a strong induction by IFN-gamma (data not shown). Among the ISGs strongly induced by IFN gamma and poorly induced by IFN alpha, we focused on that coding for IDO—an intracellular enzyme involved in the rate-limiting step in tryptophan catabolism via the kynurenine pathway and which is reportedly active against many viruses [8,9,11,17,18,19,20]. Furthermore, IDO’s immunomodulatory role makes it a promising candidate for combatting BKPyV infections.

### 3.3. The Antiviral Activity of IFN-Gamma (BKPyV Infection of Caki-1 Cells)

**Figure 3:**
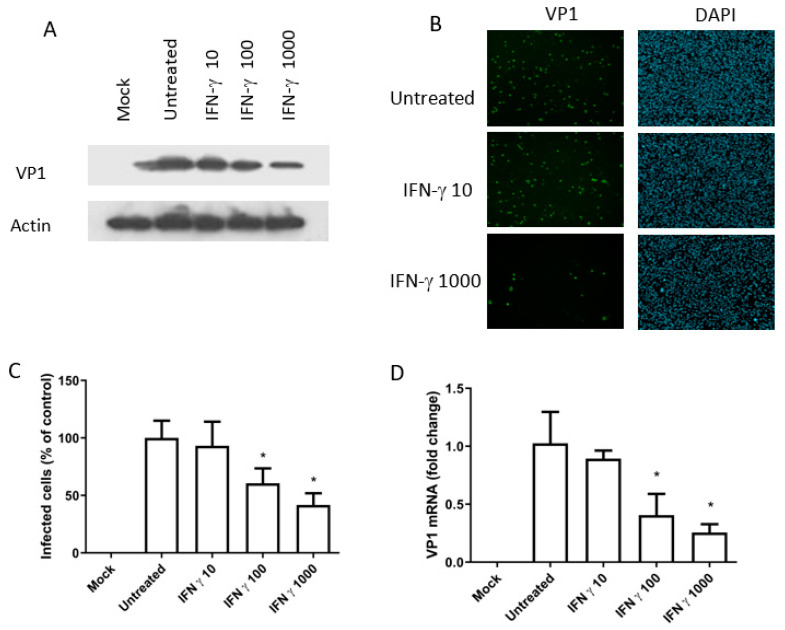
Interferon-gamma has dose-dependent activity against BKPyV in cultures of Caki-1 cells. Caki cells were treated 6 h before BKPyV infection (MOI: 0.5) with 10, 100 or 1000 IU/mL IFN-gamma (**A**) Total cellular proteins were extracted 72 h after infection. VP1 and actin were detected by Western blotting. (**B**) VP1 expression was analyzed using immunofluorescence. The cells were fixed three days post-infection and stained with monoclonal anti-BKPyV VP1 antibody (in green). The DNA was stained with DAPI (in blue). Magnification x400. (**C**) The percentage of infected cells was calculated from (**B**) by comparison with the DAPI staining. (**D**) mRNA levels of VP1 were measured using real-time quantitative RT-PCR and normalized against *GAPDH*. Each bar represents the average of three independent experiments analyzed in triplicate. “Mock” corresponds to non-infected cells and “Untreated” corresponds to cells infected in the absence of IFN. * *p* < 0.05.

To determine whether the observed antiviral effect is specific to monkey-derived Vero cells, we chose to investigate the role of IDO in human renal cell lines (Caki-1 and RPTE/TERT1 cells). In order to avoid poor compatibility between the plasmid encoding the human *IDO1* gene and the monkey cells (which might compromise IDO’s activation), we first determined whether IFN-gamma had an antiviral effect in cultures of human Caki-1 cells. To this end, Caki-1 cells were stimulated with various IFN-gamma concentrations and infected with BKPyV. Three days post-infection, total cellular proteins were harvested and VP1 expression was measured by Western blotting. Treatment with IFN-gamma was associated with significantly lower VP1 expression (relative to control experiments in the absence of IFN) in a dose-dependent manner (Figure 3A). Furthermore, an increase in the IFN dose was associated with a fall in the percentage of infected cells (Figure 3B,C). Since we had observed an influence of IFN on viral protein expression, we next determined whether IFN-gamma inhibited the production of BKPyV transcripts. Total RNA of infected cells was collected 72 h after infection. The samples were analyzed in a real-time quantitative-PCR assay for VP1 mRNA. As expected, the level of transcription inhibition was similar to that seen for VP1 protein expression 72 h after infection (Figure 3D).

### 3.4. Expression of IDO in Caki-1 Cells

**Figure 4:**
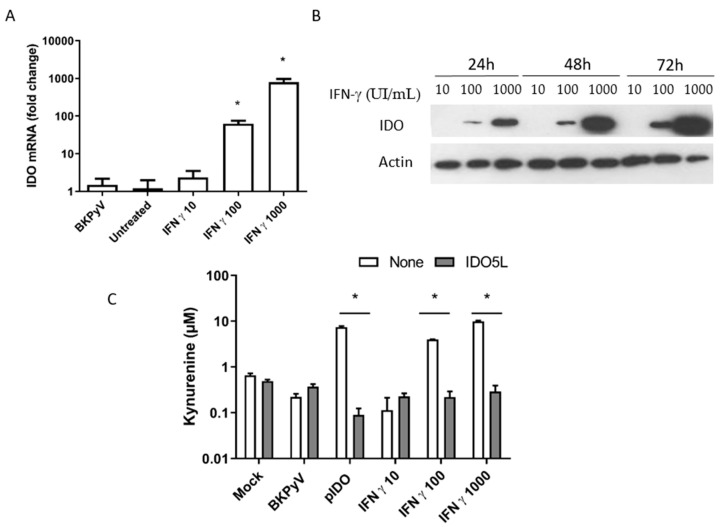
IFN-gamma promotes the over-expression of functionally active indoleamine 2,3-dioxygenase (IDO) in Caki-1 cells. (**A**) Caki cells were treated with IFN-gamma or transfected with a plasmid carrying the human *IDO1* gene. Three days after IFN treatment or transfection, the level of *IDO1* mRNA was measured using a quantitative RT-PCR assay. (**B**) For IDO protein expression, proteins were collected 24, 48 or 72 h after IFN treatment. The Western blot shows the IDO expression after treatment with 10, 100 or 1000 IU/mL of IFN. The results in A and C represent the mean ± SD of three independent experiments performed in triplicate. “Mock” corresponds to non-infected cells and “Untreated” corresponds to cells infected in the absence of IFN. * *p* < 0.05. (**C**) IDO’s functional activity and its inhibition by IDO5L at 50 µM were determined by measuring kynurenine accumulation in Ehrlich’s colorimetric reaction.

We next determined whether stimulation with IFN-gamma induced the expression of IDO in Caki-1 cells. To this end, Caki-1 cells were incubated with various IFN-gamma concentrations (ranging from 10 to 1000 IU/mL). We first monitored *IDO1* mRNA levels using a real-time quantitative RT-PCR (Figure 4A). Cells stimulated with IFN-gamma upregulated *IDO1* mRNA in a dose-dependent manner. In contrast, *IDO1* mRNA was not induced without IFN-gamma stimulation or by the virus. The expression of IDO was checked by Western blotting (Figure 4B). IDO was detected 24 h after stimulation with 100 and 1000 IU/mL IFN-gamma but not at all with 10 IU/mL IFN-gamma—even 72 h post-stimulation. These results show that IFN-gamma stimulated IDO expression in Caki-1 cells. We also measured IDO’s enzymatic activity by quantifying the production of the catabolite kynurenine at three different times after IFN-treatment (24, 48 and 72 h) and after transfection with a plasmid carrying the human *IDO1* gene. As shown in Figure 4C, our observation of dose-dependent kynurenine production confirmed that IDO is active in Caki-1 cells. The fact that IDO5L was able to block kynurenine production also confirmed that the IDO in Caki-1 cells was functional.

### 3.5. The Antiviral Effect of IDO (BKPyV Infection of Caki-1 Cells)

**Figure 5:**
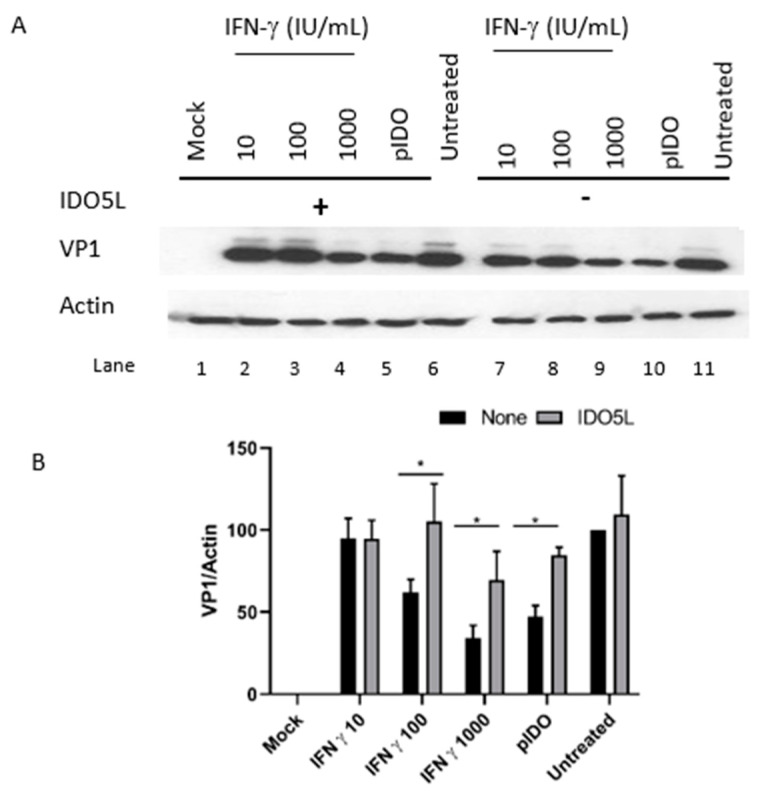
The effect of IDO on BKPyV infection. Caki cells were transfected with a plasmid carrying the human *IDO1* gene (lanes 5 and 10) or treated with various concentrations of IFN gamma (lanes 2, 3 and 4, and lanes 7, 8 and 9). IDO5L was added to 6 samples (lanes 1 to 6). The cells were then infected 24 h post-transfection, and total proteins were harvested 3 days post-infection. (**A**) VP1 and actin were detected by Western blotting. (**B**) Densitometric analysis of VP1 (normalized against actin). The results in B corresponds to the mean ± SD of three independent protein samples per condition. “Mock” corresponds to non-infected cells and “Untreated” corresponds to cells infected in the absence of IFN. * *p* < 0.05.

To assess IDO’s effects on BKPyV infection, we transfected Caki-1 cells with a plasmid encoding human *IDO1* and then infected them with BKPyV. Seventy-two hours later, we observed low VP1 expression (relative to a control experiment) on a Western blot; this confirmed IDO’s inhibitory effect on the BKPyV infection (Figure 5A, lane 10). The fact that this low expression was reversed by the addition of the IDO inhibitor IDO5L suggested that the effect was specific for IDO (Figure 5A, lane 5). To determine IDO’s role in the antiviral effect of IFN-gamma, we treated infected Caki-1 cells with IFN-gamma in the presence of IDO5L. As shown in Figure 5A (lanes 2, 3 and 4 vs. lanes 7, 8 and 9), IDO5L partially rescued the BKPyV infection. The action of IDO5L accounted for 40% of IFN-gamma’s antiviral activity (Figure 5B).

### 3.6. Antiviral Effect of IFN-Gamma and IDO in RPTE/TERT1 Cells

**Figure 6:**
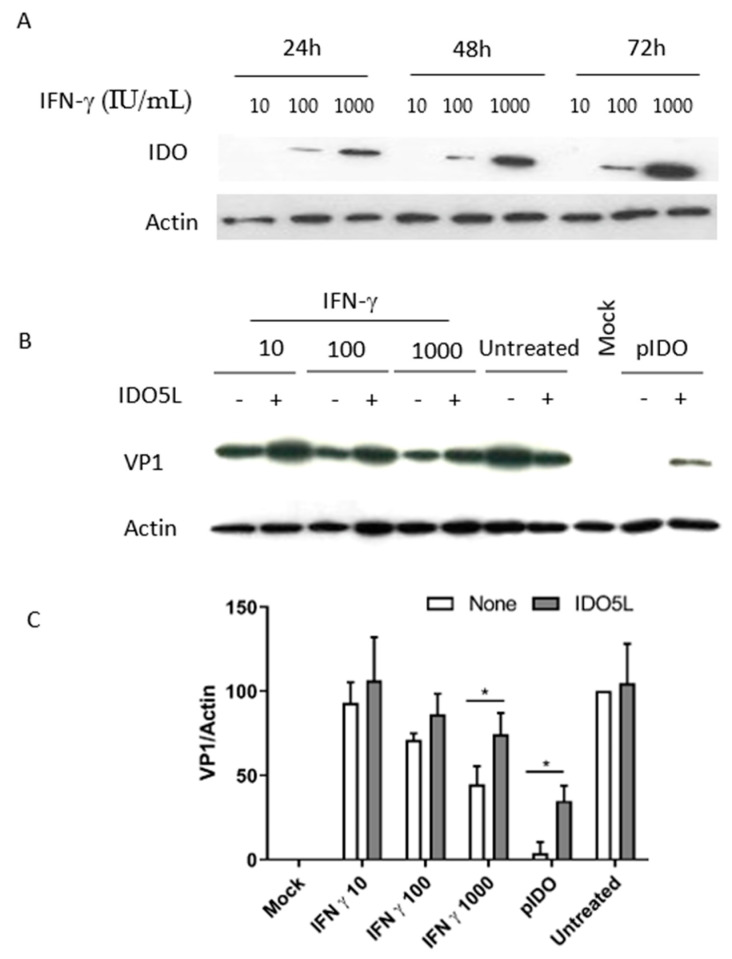
Antiviral effect of IFN-gamma and IDO with pLXSN-hTERT retroviral transfection (RPTE/TERT1) cells. RPTE/TERT1 cells were treated with IFN-gamma and collected after 24, 48 or 72 h. (**A**) Levels of IDO and actin expression were monitored by Western blotting. Infected cells were treated with 10, 100 and 1000 IU/mL IFN-gamma or transfected with a plasmid encoding human *IDO1*. IDO5L was added at the same time as IFN treatment or transfection. Total cellular proteins were harvested 3 days after infection. (**B**) VP1 and actin were detected by Western blotting. (**C**) Densitometric analysis of VP1, normalized against actin. The results in C represent the mean ± SD of three independent protein samples per condition. “Mock” corresponds to non-infected cells and “Untreated” corresponds to cells infected in the absence of IFN. * *p* < 0.05.

To confirm these results, we also performed the same experiments on RPTE/TERT1 cells. We observed the rapid induction of IDO after IFN-gamma treatment of RPTE/TERT1 cells (Figure 6A). VP1 expression was strongly reduced after cell transfection with the plasmid carrying the human *IDO1* gene (Figure 6B,C). Moreover, IFN-gamma’s antiviral effect was partially restored by the presence of IDO inhibitor IDO5L (Figure 6B,C), suggesting that IDO contributes to the effect.

## 4. Discussion

Our results demonstrated that BKPyV’s sensitivity to IFN depended on the type of cytokine. The first surprising finding was the moderate inhibitory effect of IFN-alpha on BKPyV infection. A low level of VP1 protein expression was only observed with 100 IU/mL of IFN-alpha, the VP1 mRNA expression was 40% of the positive control value at this concentration. There are two possible explanations for this low degree of sensitivity to type I IFN, which has also been described for human papillomavirus (HPV) [21]. Firstly, the IFN response (via ISG induction) might not be effective for DNA viruses. *Papillomaviridae* and *Polyomaviridae* have a common replication strategy; they use the host cell’s machinery (rather than viral polymerases) for multiplication. Secondly, these viruses might have developed ways of evading IFN’s antiviral effect. We showed here that IFN-lambda had only a small effect on the levels of early and late viral transcripts in cultures of Vero cells. Again, the same result has been described for HPV [21]. We found that type II IFN had a strong antiviral effect from a concentration of 10 IU/mL upwards, as described previously [6]. Furthermore, we confirmed that levels of VP1 and LTag viral mRNA were low when Vero, Caki-1 and RPTE/TERT1 cells were exposed to IFN-gamma.

Assetta et al. showed that BKPyV’s infection of RPTE cells was sensitive to a low concentration (10 IU/mL) of IFN beta [22]. We did not observe this effect with IFN alpha. Type I IFN alpha and beta have the same receptor (IFNAR1) and similar main activities but are not functionally redundant. Indeed, IFN beta’s affinity for IFNAR1 is 50 times higher than that of IFN alpha, which explains the associated anti-proliferative activity [23]. This anti-proliferative activity might be important for IFN-beta’s antiviral effect, since cell proliferation is a key event in BKPyV multiplication.

In view of these results, we investigated Stat1 phosphorylation. In line with the results for antiviral activity, we found that Stat1 was briefly phosphorylated after IFN-alpha treatment and lastingly phosphorylated with IFN-gamma treatment. We did not observe any Stat1 phosphorylation with IFN-lambda. These results suggest that prolonged Stat1 phosphorylation is necessary for IFN-gamma’s induction of an antiviral state.

Our present results highlighted the antiviral role of IFN-gamma in the context of BKPyV infection. Several clinical studies attest to the importance of IFN-gamma in the clearance of BKPyV infections [24,25,26]. However, IFN-gamma has also harmful effects, such as the promotion of graft rejection. We therefore sought to address IFN-gamma’s mechanism of action in this context and identify potentially involved IFN-induced proteins. Since many IFN-induced proteins could potentially be involved in this effect, we focused our attention on IDO. This enzyme was a good candidate because (i) it has already been described as being active against various viruses [8,9,11,17,18,19,20], and (ii) its immunomodulatory effect might be of value in the avoidance of graft rejection [27]. By studying two human cell lines (Caki-1 and RPTE/TERT1 cells), we demonstrated that IDO inhibits BKPyV multiplication. Moreover, the addition of an IDO inhibitor partially restored the viral infection. The antiviral contribution of IDO has been well characterized for the human respiratory syncytial virus [28]. The enzyme’s primary physiological function is to catalyze the degradation of tryptophan, which decreases the availability of this essential amino acid and produces various metabolites (including kynurenine). Tryptophan availability is fundamental for the replication of intracellular pathogens, and so this modulation appears to be IDO’s main antiviral mechanism. However, IDO’s role as an antimicrobial factor in vivo is subject to debate. In fact, IDO’s biological functions and ultimate effects (either antimicrobial or immunoregulatory) are believed to depend on the type of pathogen [29]. For example, mice models of infection have shown that IDO facilitates the elimination of *Toxoplasma gondii* but suppresses the elimination of *Leishmania major* [29]. Indeed, a genetic deficiency in IFN-gamma or IRF1 (in IFN-gamma/or IRF1/mice) and the pharmacological inhibition of IDO leads to massive mortality in mice a few days after acute infection with *Toxoplasma gondii*. In contrast, in an in vivo model of HSV1 infection, inhibition of IDO activity does not affect viral replication, virulence or induction of latency [29]. The activity of IDO also impacts the immune system. Indeed, a decrease in tryptophan levels and an increase in kynurenine levels both lead to the suppression of T cell function and thus generate an immunosuppressive microenvironment around cells that display IDO activity [29]. This immunomodulatory effect might be very valuable in the context of transplantation by both generating an antiviral effect and protecting the graft from rejection. Nevertheless, T cell inhibition might also impede the BKPyV-specific response that is thought to be particularly effective for viral clearance [25,26,27]. Furthermore, immunomodulation by IDO is also involved in decreased the anti-tumor defenses, which might be dangerous in transplant recipients. These considerations emphasize the importance of developing an in vivo model (such as an IDO/mouse) in which to study the true impact of IDO on BKPyV.
